# A missense founder mutation in *VLDLR* is associated with Dysequilibrium Syndrome without quadrupedal locomotion

**DOI:** 10.1186/1471-2350-13-80

**Published:** 2012-09-14

**Authors:** Bassam R Ali, Jennifer L Silhavy, Matthew J Gleeson, Joseph G Gleeson, Lihadh Al-Gazali

**Affiliations:** 1 Department of Pathology, Faculty of Medicine and Health Sciences, United Arab Emirates University, Al-Ain, United Arab Emirates; 2 Department of Neurosciences and Pediatrics, Howard Hughes Medical Institute, University of California, San Diego, USA; 3 Department of Pediatrics, Faculty of Medicine and Health Sciences, United Arab Emirates University, P.O. Box 17666, Al-Ain, United Arab Emirates

## Abstract

**Background:**

Dysequilibrium syndrome is a genetically heterogeneous condition that combines autosomal recessive, nonprogressive cerebellar ataxia with mental retardation. The condition has been classified into cerebellar ataxia, mental retardation and disequilibrium syndrome types 1 (CAMRQ1), 2 (CAMRQ2) and 3 (CAMRQ3) and attributed to mutations in *VLDLR*, *CA8* and *WDR81* genes, respectively. Quadrupedal locomotion in this syndrome has been reported in association with mutations in all three genes.

**Methods:**

SNP mapping and candidate gene sequencing in one consanguineous Omani family from the United Arab Emirates with cerebellar hypoplasia, moderate mental retardation, delayed ambulation and truncal ataxia was used to identify the mutation. In a second unrelated consanguineous Omani family, massively parallel exonic sequencing was used.

**Results:**

We identified a homozygous missense mutation (c.2117 G > T, p.C706F) in the *VLDLR* gene in both families on a shared affected haplotype block.This is the first reported homozygous missense mutation in VLDLR and it occurs in a highly conserved residue and predicted to be damaging to protein function.

**Conclusions:**

We have delineated the phenotype associated with dysequilibrium syndrome in two Omani families and identified the first homozygous missense pathogenic mutation in *VLDLR* gene with likely founder effect in the southeastern part of the Arabian Peninsula.

## Background

Dysequilibrium syndrome (DES) is a heterogeneous autosomal recessive genetic condition characterized by non-progressive cerebellar ataxia, cerebellar hypoplasia, moderate-to-profound mental retardation and delayed ambulation [1]. DES has been reported in American Hutterite patients [2-4] as well as in Turkish [5-7], Iranian [8], Caucasian [9], Iraqi [10] and Saudi [11] patients. DES belongs to congenital ataxia with cerebellar hypoplasia group of heterogeneous disorders that are generally characterized by motor disability, muscular hypotonia, lack of coordination and impaired motor development [12]. Quadrupedal locomotion has been described in a portion of patients with DES [5,6,10].

Using homozygosity mapping, Boycott et al. [4] localized a gene for DES to chromosome 9 and identified a 199-kb homozygous deletion encompassing the entire *VLDLR* gene in affected individuals. Subsequently, a homozygous nonsense mutation (c.1342C > T, p.R448X) was identified in an Iranian family [8] and a nonsense mutation (c.769C > T, p.R257X) and a single nucleotide deletion (c.2339delT, p.I779fsX3) were identified in Turkish families [5,6]. More recently, Kolb et al. [13] have identified a microdeletion encompassing exons 2, 3 and 4 of the *VLDLR* gene in a Turkish family with pachygyria and pontocerebellar atrophy. In addition, mutations in *CA8* gene have been implicated in a closely related syndrome that is characterized by ataxia and mild mental retardation with predisposition to quadrupedal gait [10]. Furthermore, it has been recently shown that mutations in the *WDR81* gene are the underlying cause of cerebellar hypoplasia and quadrupedal locomotion in a consanguineous Turkish kindred [7].

VLDLR is a member of the LRP (LDD-related protein) family, which also includes apolipoprotein E receptor 2 (ApoER2), the LDL receptor (LDLR), LRP and Megalin [14]. Members of this family of proteins are structurally related multifunctional cell-surface receptors that mediate endocytosis of extracellular ligands. In addition, these receptors play key roles in a wide range of physiological processes including the regulation of lipid metabolism, protection against atherosclerosis, neurodevelopment, signaling and transport of nutrients and vitamins [14,15]. In particular, VLDLR is involved in the reelin signalling pathway, which guides neuroblast migration in the cerebral cortex and cerebellum [16,17].

In this article, we report the identification of a novel founder missense mutation in *VLDLR* gene in two consanguineous unrelated Omani families with Dysequilibrium syndrome, VLDLR type (CAMRQ1, OMIM 224050).

## Methods

Families were evaluated clinically for signs and symptoms related to cognitive impairment and ataxia with onset in the first year of life. Full general and neurological assessments and standard brain MRI were performed to document any structural alterations. The study was approved by the Ethics Committees at both institutions and informed consents for participation in the study were obtained from the participants or, where participants are children, were obtained from a parent. Peripheral blood samples were collected on all genetically informative members of both families and DNA was extracted using Qiagen reagents according to the manufacturer’s instructions.

### DNA methodologies

For Family 1, a 5 K whole genome linkage SNP scan was performed using the Illumina Linkage IVb mapping panel [18] and analyzed with easyLinkage-Plus software [19]. The *VLDLR* candidate gene was sequenced using primers designed to amplify each of the coding exons and splice sites using the ExonPrimer software ( http://ihg.gsf.de/ihg/ExonPrimer.html) as described [8]. For Family 2, exome sequence was generated from individual IV-8, by exome capture with the Agilent SureSelect Human All Exome 50 Mb kit, sequenced on an Illumina HiSeq2000 instrument, resulting in ~95% recovery at >10x coverage. GATK software [20] was used for variant identification. These variants were used to identify identity-by-descent blocks with the online software HomozygosityMapper [21], set to analysis of blocks containing 200 markers and only flag blocks > 100 markers in a row. Variants were annotated using SeattleSeq ( http://snp.gs.washington.edu/SeattleSeqAnnotation/) to identify homozygous potentially deleterious variants. For SNP genotyping, informative markers from Family 1 were selected based upon the previous 5 K SNP genotyping results surrounding the VLDLR gene at 100,000-1,000,000 bp intervals on chromosome 9 and amplified in one affected individual from each Family and from a healthy control individual. PCR amplification was carried out using a standard touchdown PCR protocol. After successful amplification, PCR products were sequenced on an ABI 3730 sequencer (Applied Biosystems, Foster City, CA, USA) and analyzed using Sequencher software (Genecode, Inc).

## Results

### Phenotypic details

#### Family 1

**Figure 1:**
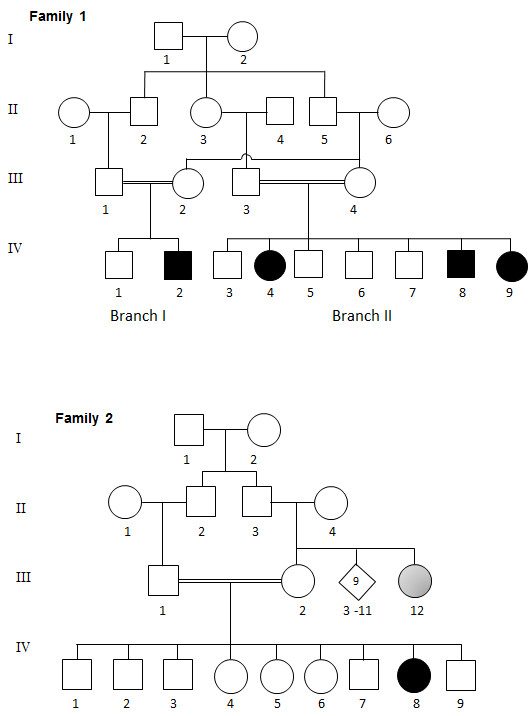
**Pedigrees of the consanguineous Emirati families of Omani origins.** Both families display typical recessive modes of inheritance. Double bar represents consanguinity; diamond represents several siblings of varying sex. Affected members are represented by filled symbols and the gray symbol as possibly affected status.

**Figure 2:**
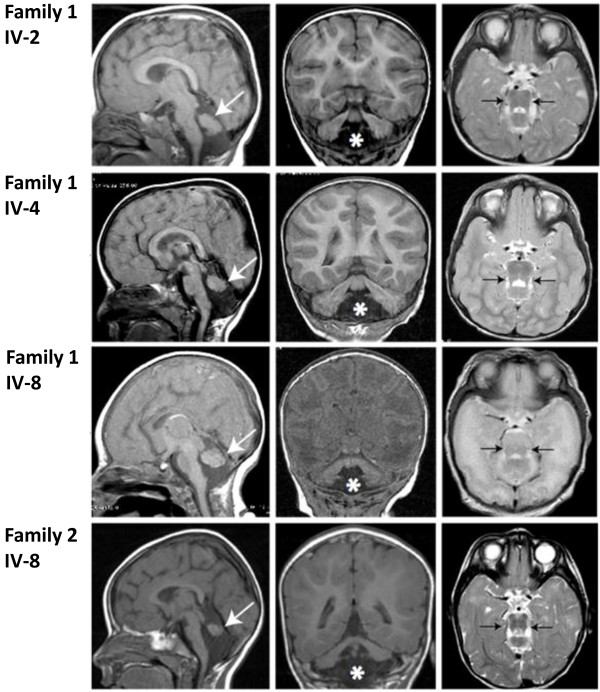
**Brain MRI of affected individuals from Families 1 and 2.** Left: midline parasaggital T1-weighted images showing absent inferior vermis, and hypoplastic inferior-cerebellum (arrows). Middle: coronal T1-weighted images showing neocortical pachygyria and mega cisterna magna (*). Right: Axial T2-weighted images showing typical square-shaped midbrain-hindbrain junction morphology in all patients (arrows).

This family is from UAE of Omani origin and is highly inbred. There are 4 affected children in two branches (Figure 1 Family 1). Their ages range from 3 to 14 years. All presented with hypotonia with exaggerated reflexes and delayed developmental milestones in the first year of life. In addition, all had truncal ataxia and none achieved walking independently. The elder siblings, however, could walk with help and their gait was bipedal. Moderate mental retardation was present in all of them. One of the four affected children had left side strabismus otherwise ophthalmological examination was normal. All had no speech development. There were no seizures or dysmorphic features in any of the children (Table 1). Their weights (10-50%), heights (5-25%) and head circumferences (10-25%) were in the normal ranges. Brain MRI demonstrated diffuse cortical pachygyria without significant white matter signal abnormality, as well as characteristic cerebellar hypoplasia but intact pontine volume (Figure 2). The cerebellar folia were almost completely absent, and there was vermis greater than hemispheric hypoplasia. The pons showed a typical square shape (Figure 2).

**Table 1:** **Summary of the clinical and molecular features of the reported cases with Dysequilibrium Syndrome- *VLDLR *type**

	**Boycott et al. 2005 [**[4]**]**	**Moheb et al. 2008 [**[8]**]**	**Turkman et al. 2008 [**[6]**]**	**Ozcelik et al. 2008 [**[5]**]**	**Ozcelik et al. 2008 [**[5]**]**	**Boycott et al. 2009 [**[9]**]**	**Kolb et al. 2010 [**[13]**]**	**This report Family 1**	**This report Family 2**
**General**									
**No. of patients**	**10**	**8**	**3**	**5**	**3**	**1**	**2**	**4**	**1**
Ethnicity	Hutterite	Iranian	Turkish	Turkish	Turkish	Caucasian	Turkish	UAE (Omani)	UAE (Omani)
Consanguinity	+	+	+	+	+	-	+	+	+
VLDLR mutation	Homozygous deletion of entire gene	Homozygous c.1342C>T (p.R448X)	Homozygous c.2339delT (p.I780TfsX3)	Homozygous c.769C.T (p.R257X)	Homozygous c.2339delT (p.I780TfsX3)	Compound heterozygous c.1561G>C + c.1711-1712dupT (p.D521H + p.Y571LfsX7)	Homozygous deletion of exons 2, 3, 4 and parts of exons 1 and 5	Homozygous c.2117G>T (p.C706F)	Homozygous c.2117G>T (p.C706F)
**Clinical**									
Mental retardation	Moderate-Profound	Moderate-Profound	Moderate-Profound	Profound	Profound	Developmental Delay	Developmental delay	Moderate	Moderate
Hypotonia in infancy	?	?	?	-	-	+	+	+	+
Ambulation	Delayed	-	Delayed	Delayed	Delayed	Delayed	Delayed	Delayed	Delayed
Gait	Bipedal	Bipedal	Quadrupedal	Quadrupedal	Quadrupedal	Bipedal	Bipedal	Bipedal	Bipedal
Truncal ataxia	+	?	+	+	+	+	+	+	+
Dysarthric Speech	+	?	+	+	+	?	+	No Speech	No Speech
Seizure	+/-	-	?	+/-	-	-	-	-	-
Strabismus	+/-	+	?	+	+	-	-	+/-	+
**Neuroimaging**									
Absent inferior vermis	+	?	+	+	+	+	+	+	+
Hypoplastic inferior-cerebellum	+	?	+	+	+	+	+	+	+
Simplified cortical gyri	+	?	+	+	+	+	+	+	+

#### Family 2

This is also an inbred UAE family of Omani origin (Figure 1 Family 2) and they deny any knowledge of being related to Family 1. The couple has 9 children with only one affected child. In addition, the mother’s sister who is 40 years old now (III-12) has moderate-severe mental retardation and is unable to walk although we were unable to fully evaluate her as she lives in Oman. The affected child (IV-8) was the product of normal pregnancy and delivery. She was noted to have left side strabismus and was reported to be very quiet in the first few months of life. Evaluation at three months revealed, in addition to the left side strabismus, severe hypotonia with exaggerated reflexes. She had delayed developmental milestones and was unable to walk unaided at the age of six years. She had no speech development at all (Table 1). Her weight (10th), height (>10th) and head circumference (10th) were all normal. Brain MRI demonstrated cortical pachygyria, cerebellar hypoplasia, and square-shaped pons, strikingly similar to the findings in Family 1 (Figure 2).

### Identification of a pathogenic missense mutation

**Figure 3:**
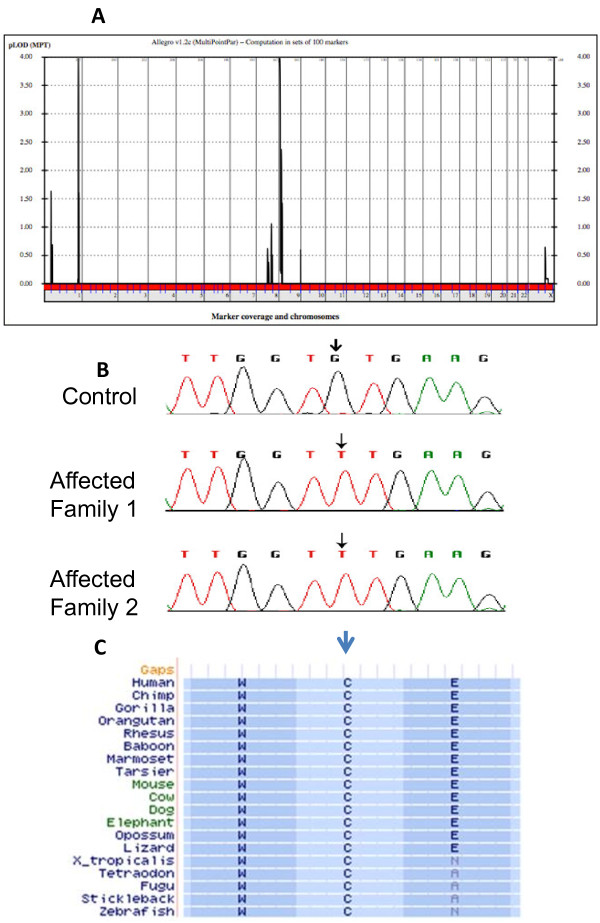
**Molecular characterization of Families 1 and 2.A**: Linkage plot for Family 1 with major peaks of pLOD score 4 achieved, both for chr. 9 and chr. 1. x-axis represents chromosomal position, y-axis represents pLOD score. **B**. Sequence chromatogram from control and affected individuals from Families 1 and 2, with mutant G to T residue at position chr9:2650382 (c.2117 G > T). **C**. Conservation of wild-type encoded C residue across annotated vertebrate species.

In order to identify the genetic cause of disease in Family 1, we performed linkage analysis using a 5 K SNP linkage panel on each of the members of generation III and IV, and identified one linkage peak on chromosome 9 (Figure 3A) that overlapped the *VLDLR* gene locus, known to cause DES [8]. An additional linkage peak on chromosome 1 did not contain genes known to cause neurological disturbances. Direct sequence of each of the 17 exons including canonical splice sites from individual IV-4 revealed a single probable pathogenic mutation (Figure 3B) in exon 15 of the *VLDLR* gene at base position chr9:2650382 G > T according to the hg19 build of the human genome. For Family 2, because we had no reason to suspect *VLDLR* gene mutations over the other DES genes or potentially novel causes, exome sequencing of the affected child from generation IV (IV-8) was performed and revealed the same chr9:2650382 G > T mutation (Figure 3B). This alteration resulted in a c.2117 G > T nucleotide change and a p.C706F amino acid substitution in the transcript NP_003374.3. This change resulted in altering a cysteine residue that is conserved across all of vertebrate evolution (Figure 3C). The variant produced a Grantham score of 205 and a PhastCon score of 1.0 [22,23]. In addition, the mutation was predicted by PolyPhen-2 [24] to be probably damaging with a score of 1.0, which is the highest score possible. Furthermore, the mutation was not identified in dbGaP nor was it encountered in over 1000 exome sequence analyses from Middle Eastern patients with other diseases in our in-house database.

### Identification of a common founder haplotype in Family 1 and 2

The finding that the two families shared a common genetic mutation in the *VLDLR* gene suggested the possibility of a common founder mutation. In order to test for a shared affected haplotype, we designed PCR primers at spaced intervals surrounding the *VLDLR* mutation, which were selected to be informative in Family 1 from the previous 5 K SNP analysis. Amplification from Family 1 IV-4 and Family 2 IV-8 showed PCR products of the predicted sizes for all amplicons, and sequence analysis of each SNP was in full agreement with Family 1 previous SNP analysis. Family 2 genotyping showed identical homozygous calls for SNPs rs1535842, rs1331829, rs729367, rs1455175, from base position 2,212,796-3,796,061 on chromosome 9, or a distance of approximately 1,500,000 base pairs (Table 2). This haplotype was not encountered in any of ~100 UAE individuals on whom whole genome SNP results were reviewed in our lab, suggesting that the mutation occurred on a uniquely shared haplotype and segregated in the two families.

**Table 2:** **Genotyping from Family 1 and Family 2 surrounding the *VLDLR *mutation suggests a common ~1,500,000 base pair haplotype inherited by affected individuals in both families, between base position chr9:2,212,796 and chr9:3,796,061**

**RS Number or gene**	**Base Position (chr 9, hg19)**	**Reference**	**Variant**	**Control**	**Family 1**	**Family 2**
rs6474795	1399674	A	T	T/T	T/T	T/T
rs1412256	1464067	G	C	C/C	G/G	C/C
rs1535842	2212796	C	T	C/T	T/T	T/T
rs1331829	2382952	G	T	G/G	G/G	G/G
rs729367	2600253	T	C	T/C	T/T	T/T
*VLDLR*c.2117 G > T	2650382	G	n/a	G/G	T/T	T/T
rs1455175	3796061	G	C	C/C	C/C	C/C
rs4131424	4335668	G	A	G/A	G/A	G/G
rs7851353	4411383	A	G	A/G	G/G	G/G
rs913258	4877246	C	G	G/G	C/C	G/G

## Discussion

DES is genetically and phenotypically heterogeneous condition that combines autosomal recessive, non-progressive cerebellar ataxia and mental retardation with or without quadrupedal locomotion. This condition has three types: CAMRQ1 (OMIM 224050), CAMRQ2 (OMIM 610185) and CAMRQ3 (OMIM 613227). CAMRQ1 has been characterized by cerebellar hypoplasia and mental retardation with or without quadrupedal locomotion and been associated with mutations in *VLDLR* gene [4-6,8,13]. CAMRQ2 and CAMRQ3 have been associated with mutations in *CA8* and *WDR81* genes, respectively [7,10,11]. In this report, we describe the first homozygous missense mutation (c.2117 G > T; p.C706F) in VLDLR to cause DES without quadrupedal locomotion in two unrelated Omani families from UAE. Genotyping data indicated that the mutation has the same haplotype in the two families strongly suggesting a founder effect in the southeastern part of the Arabian Peninsula.

**Figure 4:**
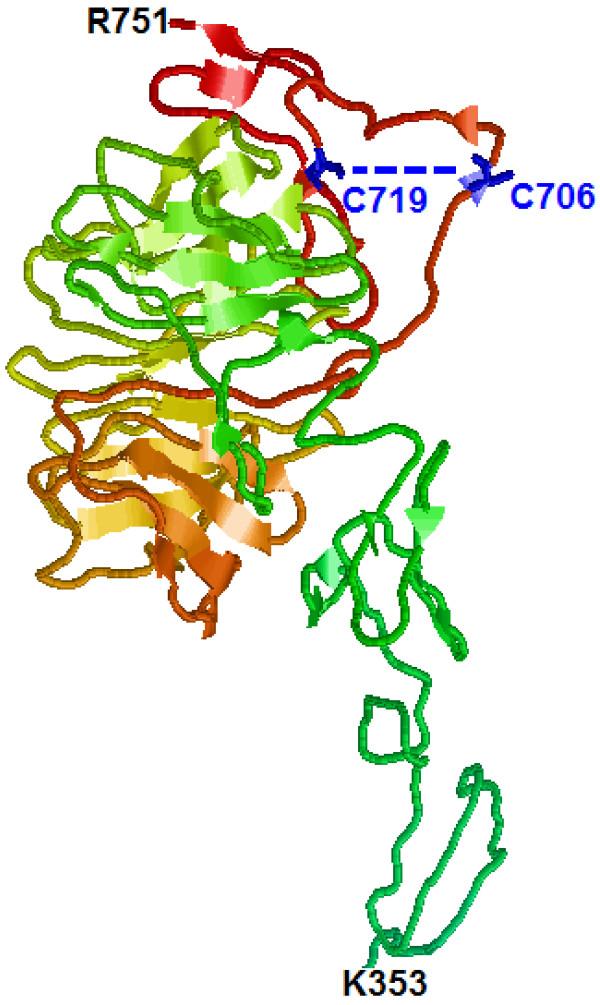
**A model of the predicted structure of R353-K751 residues of the human VLDLR.** The model was constructed using the Swiss Model program ( http://swissmodel.expasy.org) with the full length sequence of the human VLDLR protein. The model has been visualized using RasMol ( http://rasmol.org). According to the UniProtKP database, cysteine 706 is predicted to form a disulfide bond with cysteine 719 (Blue colored) ( http://www.uniprot.org/uniprot/P98155).

The mutation is located in the extracellular EGF-like 3 domain of the VLDLR protein. The cysteine residue at position 706 is highly conserved and is predicted to be involved in a disulfide bond with cysteine 719 ( http://www.uniprot.org/uniprot/P98155; see Figure 4 for the predicted model of the VLDLR domains harboring the Cys706) and therefore disruption of this residue is likely to result in misfolding of the protein, its retention in the endoplasmic reticulum and degradation by the ER-associated protein degradation machinery (ERAD) [25,26]. This in turn will lead to loss of the VLDLR protein function. Degradation by ERAD has been extensively documented for many of the LDLR missense mutations [27]. VLDLR and LDLR belong to the same LRP (LDD-related protein) family proteins [14].

The phenotype (clinical and neuroimaging) in DES, VLDLR type is highly specific. Affected individuals demonstrate significant truncal ataxia and either learn to walk late, or never achieve independent ambulation. All reported individuals with this disorder had intellectual impairment ranging from moderate to profound. Those who were able to communicate had dysarthria. The gait could either be bipedal or quadrupedal. This variability in the gait has been attributed to the complex interaction between environmental factors and the cerebellar malformation and not the type of causative mutation [13]. Other variable features reported in this disorder include strabismus, seizures, short stature and pesplanus. Brain MRI findings include hypoplasia of inferior portion of the cerebellar vermis and hemispheres, simplified cerebral gyration with small brain stem and pons [4-6,8,13]. All affected children in this report had typical features of this disorder. All had delayed developmental milestones with moderate mental retardation, truncal ataxia, and lack of speech while strabismus was present in some of them. The gait was bipedal in those who learnt to walk (Table 1). Brain imaging showed typical abnormalities in all the affected children including cerebellar hypoplasia and diffuse cortical pachygyria (Table 1, Figure 2).

Kaya et al. [11] have recently described the phenotypic spectrum and molecular analysis of three related families from northern Saudi Arabia, a neighboring country to Oman and UAE, with cerebellar ataxia, mental retardation and DES. Despite some phenotypic variability among the affected individuals in the three families, they all shared a homozygous missense mutation (c.484 G > A; p.G162R) in *CA8* gene indicating the possible involvement of modifier factors. This mutation was different from the one reported (c.298 T > C; p.S100P) in Iraqi patients [10] excluding the possibility of a founder effect. The Saudi and Iraqi families shared mild cognitive impairment, variable degree of cerebellar ataxia, absence of seizures and absence of dysmorphism. However, the patients in the Saudi families did not exhibit the quadrupedal locomotion that was present in the Iraqi patients. This is reminiscent to the *VLDLR*-associated DES, where some patients with *VLDLR* mutations exhibited quadrupedal locomotion while others did not. It was argued that environmental factors are the underlying causes of this phenotypic variation [4,5,8,9,28-30]. The affected individuals in our two families share a common mutation and founder haplotype, suggesting that it should be possible to provide targeted testing for this mutation in patients from this population.

## Conclusions

Our data expand the mutation spectrum of dysequilibrium syndrome, *VLDLR* type, and demonstrate the presence of a missense founder mutation in patients with this syndrome in the southeastern part of the Arabian Peninsula.

## Competing interests

We declare that there are no competing interests for any of the authors on this article.

## Authors’ contributions

Study design: BRA, JGG and LA. Clinical evaluation of patients and sample collection: LA, and JGG. Generation and analysis of data: BRA, JLS, MJG, and LA. Preparation of the manuscript: BRA, JLS, LA and JGG. All the authors read and approved the final manuscript.

## Pre-publication history

The pre-publication history for this paper can be accessed here:

http://www.biomedcentral.com/1471-2350/13/80/prepub
